# Young lives disrupted: gender and well-being among adolescent Syrian refugees in Lebanon

**DOI:** 10.1186/s13031-017-0128-7

**Published:** 2017-11-14

**Authors:** Jocelyn DeJong, Farah Sbeity, Jennifer Schlecht, Manale Harfouche, Rouham Yamout, Fouad M. Fouad, Seema Manohar, Courtland Robinson

**Affiliations:** 1 Faculty of Health Sciences, American University in Beirut, Beirut, Lebanon; 2 Women’s Refugee Commission, New York, USA; 3 Save the Children, Fairfield, CT USA; 4 Department of Population, Family and Reproductive Health, Johns Hopkins Bloomberg School of Public Health, Baltimore, MD USA

**Keywords:** Refugee, Adolescent, Youth, Gender, Reproductive health, Well being

## Abstract

**Background:**

The conflict in Syria that began in 2011 has resulted in the exodus of over 5 million Syrian refugees to neighbouring countries, with more than one million refugees currently registered by UNHCR in Lebanon. While some are living in tented settlements, the majority are living in strained conditions in rented accommodation or collective shelters in the Bekaa Valley next to Syria. Adolescents are particularly vulnerable in any crisis. In 2013–4, the American University in Beirut in collaboration with the Women’s Refugee Commission, Johns Hopkins and Save the Children, sought to understand the specific experiences of very young adolescents, those 10–14 years of age, in this protracted crisis context.

**Methods:**

The study was conducted in 2014 in Barelias and Qabelias – two urban areas located close to each other in the Beka’a valley that has a large concentration of Syrian refugees. Focus group discussions (FGDs), including community mapping and photo elicitation, were conducted with 10–12 and 13–14 year old Syrian refugee adolescents, in order to obtain information about their experiences and perspectives. FGDs were also implemented with 15–16 year old Syrian refugees and separately also with adult refugees, to consider their perspectives on the needs and risks of these adolescents.

**Results:**

A total of 16 FGD (8 for each sex, with 6–9 participants in each) were conducted in Arabic across the two sites, with 59 female participants and 59 male participants. The experiences and risks faced by these adolescents were significantly impacted by economic strain and loss of educational opportunities during displacement, and only a minority of adolescents in the study reported attending school. Additionally, on-going protection risks for girls were felt to be higher due to the crisis and displacement. In Lebanon this has resulted in increased risks of child marriage and limitations in mobility for adolescent girls. Adolescents, themselves expressed tensions with their Lebanese counterparts and feared verbal attacks and beatings from school-aged Lebanese male youth.

**Conclusions:**

Families and adolescents have been dramatically affected by the conflict in Syria, and the resulting forced displacement. The loss of educational opportunities is perhaps the most significant effect, with long-term devastating outcomes. Additionally, the futures of Syrian girls are deeply affected by new protection concerns, particularly as they are exposed to an unfamiliar and more liberal society in Lebanon. Child marriage and limitations in their mobility – particularly for girls - are presented by families as coping strategies to these risks. Programming is needed to ensure sustained education access for all adolescents, and to educate very young adolescents and their parents on managing their own health and well-being, given the multiple strains. More effort is needed to encourage positive interaction between adolescent Lebanese and adolescent Syrian refugees.

## Background

The flow of Syrian refugees to Lebanon represents a humanitarian crisis that the country is ill-prepared to address and comes after successive waves of refugees historically following conflicts in the region, including Palestinian and Iraqi refugees. In the spring of 2014, when the study reported here was conducted, Lebanon was hosting 997,251 registered Syrian refugees [1]. As of mid-2017, there are over one million refugees registered with the United Nations High Commission for Refugees (UNHCR) [1], although the interim figures had been even higher before the significant flow of refugees to Europe began [1]. Syrian refugees represent almost a quarter of the host population of Lebanon at 4,470,852 [2].

There are no official refugee camps for Syrian refugees in Lebanon; while some live in informal tented settlements, most live in and among the local population in small settlements and often in substandard housing in urban neighborhoods, aided by a common language and culture with the host population. Programming to address the health and development needs of refugees has been challenging as a result of this dispersion of the refugee population. Unregistered Syrians in particular are reported to face many obstacles in accessing health services [3–5].

Educational access has improved for Syrian refugees since September 2015, driven by a major Ministry of Education and Higher Education (MEHE) and UNICEF campaign entitled “Back to School”, which enrolls refugee children in public schools throughout the country. However, there remain significant barriers to education and dropout is high among Syrian refugees. A report by the Overseas Development Institute (ODI) on Syrian refugees in Lebanon found that the proportion of children not in school rises considerably with age, with a reported 64.29% of Syrian refugee children 6–14 years old out of school and 92.26% of children 15–18 years old out of school in 2014 [6]. The major effort to increase educational enrolment among Syrian refugees has been at the elementary level, leaving many older adolescents lacking access. An evaluation of the impact of the “Back to school” campaign led by the Ministry of Education and Higher Education in 2016 -- an unpublished study by Saint Joseph University -- surveyed 914 Syrian refugees from different regions in Lebanon and found that while about 70–80% of 8–12 years old children were enrolled in public schools, only 25.5% are enrolled at the age of 15 and 10.5% at the age of 16.

In general, adolescence is a critical period during life with particular vulnerability in the case of displacement [7]. Although there have been many studies on the situation of Syrian refugees in Lebanon, only a few studies have examined adolescent Syrian refugees in particular and the available evidence does not differentiate adolescents by age group. As of May 2015, 13% of the total Syrian refugee population consisted of individuals ranging in age between 12 and 17 years old [1] (a more detailed age breakdown not available). A situation analysis of adolescent Syrian refugees conducted in 2013–14 highlighted the potential long term negative effects of the living conditions of Syrian refugees and the fact that Syrian refugees lack legal permission to work in Lebanon [8]. This humanitarian crisis has altered every aspect of their daily life; young people reported a decrease in the “standards of personal care”, an increase in family tensions, a higher level of school drop-out (94% of young refugees between the age of 15 and 24 were not in formal education) and feelings of lacking safety in Lebanon [8]. The lives of young girls have been particularly affected by this humanitarian crisis, due to heightened fears about safety. Moreover, rates of child marriage appear to be increasing, with estimates ranging from 18% to 23% in some studies [8] compared to about 17.3% nationally in Syria before the crisis (as measured by the proportion of women aged 20–24 who married before the age of 18) [9].

Regarding the health of young people, a multi-agency assessment among adolescent Syrian refugees in Lebanon in 2014 found that 66% of sampled females and 63% of sampled males aged between 15 and 18 years old did not know about contraceptive methods, and 18% of married youth had little to no knowledge about contraception [8]. This study also identified the detrimental effects of the Syrian refugee crisis on young people’s mental health, noting that an alarming rate of 41% reported having thought about committing suicide [8]. An additional rapid needs-assessment conducted by the Child Protection in Emergencies Working Group in 2013 found an increased prevalence of child marriage and child labor as coping mechanisms to deal with the hardships faced by Syrian refugees in this crisis and highlighted the lack of access to information and health services for children [10].

### Study on adolescent Syrian refugees in Lebanon

As part of a multi-country study examining the needs and risks of very young adolescent in three humanitarian contexts including Somali refugees in Ethiopia and migrants from Myanmar in Thailand, a team from Lebanon undertook a qualitative study on the health needs and risks among very young adolescents aged 10 to 14 from Syria. The research team represented a partnership between the International Non-government Organization (INGO), Save the Children that had scaled up its response to the humanitarian crisis in Lebanon, the Faculty of Health Sciences at the American University of Beirut, the Women’s Refugee Commission and Johns Hopkins Bloomberg School of Public Health (JHSPH).

## Methods

The study team selected two sites, Barelias and Qabelias, both situated in the Lebanese Governorate of Beka’a. These are known to host a large population of Syrian refugees owing to their proximity to the Syrian border, and the historically developed commercial and family relations with Syria. The two towns were chosen because Save the Children was active in this area, and had established partnerships with the health centers that were providing services to adolescents in these areas.

### Study setting

The distribution of Syrian refugees is variable across sub-regions and the Beka’a valley alone hosts almost third of the total number of refugees in Lebanon (370,830 as of January 2016 [1]; almost 14% of them aged 12–17 years old [1]). An already underprivileged region before the impact of the Syrian conflict, the governorate of Beka’a has limited resources and as elsewhere in Lebanon host communities are burdened by the influx of refugees and compete for employment opportunities. Although the distance between the two towns, Barelias and Qabelias does not exceed 11 km, Syrian refugees experience different living conditions in these two locations. Refugees living in Barelias are more integrated into the host community, most live in apartments, and have more access to health and social services. They are mostly located at a proximity to public and private schools, informal vocational centers, the municipality headquarters and the primary health care center. Additionally, many Syrian children attend school in Barelias and their parents are more integrated into the local labor market. Qabelias, by contrast, is surrounded by spontaneous tented settlements which are dispersed and located away from the residential and commercial center of the town, most refugees live in unfinished buildings, and are more socially excluded (Personal communication Maha Haidar, International Orthodox Christian Charities health officer in Bekaa). Many families living in Qabelias’ tented settlements work as agricultural labourers starting at a very young age as an exchange for the fees for renting their tents. In addition, refugees in Qabelias face restricted mobility as compared to that of Barelias due to the area’s larger size and mountainous topography. In both sites, Syrian refugees come from different parts of Syria, extending from the south of Syria, Deraa, to the north, and having varying urban and rural backgrounds.

### Training of research team

Prior to data collection, a faculty member from JHSPH trained the Lebanon data collectors on the range of qualitative research methodologies that had been chosen by the multicountry team: focus group discussions (FGDs) inclusive of community mapping and photo elicitation interviews (PEI).

### Study participants

With the assistance of recruiters from the local Lebanese community, 118 participants, each from a different family, were selected following purposive sampling by age from Syrian households within neighborhoods with a high concentration of Syrian refugees. They were invited to attend age-specific focus groups at a subsequent date. Eligibility criteria for participation in the study were adolescents aged 10–14 or 15–16, or parents 35–45 years old, apparently cognitively functional, and generally healthy. Some difficulties were experienced in recruitment in identifying individuals who would be willing to come to the centers allocated for the FGDs. The desired number of participants was eventually reached, however, and once participants arrived at the centers and gave their oral consent, none refused or dropped out of the study. All participants were informed about the study objectives and provided consent to participate in the focus groups.

### Data collection

Data collection took place in the spring of 2014.

### Focus group discussions

A total of 16 FGD (8 for each sex, with 6–9 participants in each) were conducted in Arabic across the two sites, with 59 female participants and 59 male participants. The focus groups were convened according to the age of participants (10–12 years old, 13–14 years old, 15–16 years old, 35–45 years old). The groups were segregated by sex and the facilitators were sex-matched with the participants to increase the chances of addressing culturally sensitive issues. Focus group discussions took place at the Islamic Health Center in Qabelias and at the Municipality in Barelias that hosts the health center in this village.

The adolescent focus group topic guides broadly covered household life, school, religious influence and institutions, places to socialize, perceptions of safety, perception of health and health services, information and perceptions about sexual and reproductive health, differential responsibilities of men and women, and their quality of life in Lebanon generally after their displacement.

The topic guide for the focus groups with adults focused on definitions and expectations of the different stages of adolescence, changing norms with regard to transitions, marriage and coming of age practices, adult perceptions about adolescent health, the roles of parents and their relationships with young adolescents in their community, pre- and post-displacement.

Save the Children observers attended the focus groups for two primary purposes - first, to learn from the discussion with the aim of informing their programs, and second, at the conclusion of each focus group, to provide participants with information about adolescent sexual and reproductive health programming offered in the area.

### Community mapping and photo elicitation

These two methods, which were used during FGD with younger adolescents, have been found to elicit more detailed discussion and more focused engagement, especially among younger research participants [11–13].

*The community mapping exercise* consisted of asking FGD participants 10–14 years of age, to draw collectively a map of their neighborhood, and indicate places significant in their lives (school, playground in the park, health centers, safe and unsafe places, places of worship, places to socialize). These maps were used to extract information about community living conditions and health. Maps drawn by younger adolescents were discussed during the FGDs with 15–16 years old boys and girls to seek their input on the perspectives of their younger peers.

*Photo Elicitation Interviewing* employs photographs of locally relevant places, activities, and themes— to elicit discussion [11, 12]. The themes represented in the photographs were decided on by the international study team and included gender roles, violence, early marriage and family life. The Lebanese team collaborated with Save the Children to identify photos from Lebanon that were culturally relevant and suitable for use in the study.

### Data analysis

The FGDs were all recorded with consent except for two where participants did not consent to recording and detailed notes were taken. They were then transcribed verbatim in Arabic by the facilitators and the note takers and the observers submitted their observation reports. Both the detailed notes as well as the verbatim transcripts were included in the analysis. Community maps drawn by the 10–12 and 13–14 years old groups were photographed and stored for analysis.

A three-day data analysis workshop was convened in Lebanon during which participants from the American University of Beirut, JHSPH and Save the Children (who had been involved in the data collection) read and translated the transcripts, and identified the themes related to health, security, education, family and household life, as narrated by children and adults during the FGDs. While the themes explored in the focus groups were similar across all three countries, findings below focus on the themes that emerged as particularly salient in Lebnaon.

Transcripts were translated, coded and served as the primary source of research data. Using thematic analysis, the coded transcripts were used to generate themes and subthemes. Accordingly, these themes were used to build a matrix for analyzing the findings from each of the conducted focus groups. This matrix was the structure where the collected data corresponding to each theme was inserted. The data collected from each category (girls, boys, women and men) were grouped in a spreadsheet under different themes. Analysis revealed recurrent ideas, similarities and differences among age groups and gender from the two sites.

## Results: Findings on key themes

### Education -- values and barriers

Participants – whether adolescents or adults– expressed a strong value of education. Several participants spoke of education as a means of building their future life and “making your dreams come true.” One girl shared her experience of lost educational opportunity and what that means for her:

“When we left the house [Syria] I was 13 I didn’t think it’s a big deal and I continued to play where we went. But now I think about what happened to us. I could have been growing up in my country and I could have been in school grade 10. I was a good student and I wanted to go to college but instead I’m working here in a workshop. Now when I think of this I feel bad and sad we lost everything.” (Qabelias, female 15-16 years)

Although non-formal education initiatives had recently proliferated in Lebanon, adolescent respondents were aware that such programs would not provide a needed diploma. Multiple barriers, including costs, perceived mistreatment of Syrian children in Lebanese schools and poor quality of education, as well as a different and unfamiliar curriculum further restricted enrolment. The curriculum in Syria is taught in Arabic, whereas the Lebanese curriculum includes Arabic, French and English depending on the school. Adolescents aged 13–14 years also mentioned repeatedly poor treatment of Syrian students by Lebanese teachers.

Cost was cited by adults as the most frequent barrier to school attendance. There was widespread awareness among all participants whether adult or adolescent that the recent imposition of fees of $300 to attend school, even public schools, was restrictive. Respondents – both adult and adolescent – also stressed negative experiences of Syrian children in Lebanese schools. In particular boys expressed feelings of social exclusion and bullying from Lebanese school-children, as indicated by this statement in a Barelias FGD:

“Lebanese kids wait for us till we leave school and they hit us.” (Barelias, male 13-14 years)

Parents of girls and girls themselves also described incidents of being bullied or treated badly by teachers or classmates. Some girls also spoke about mistreatment by teachers. For example, a girl stated:

“If you didn’t do anything they still come at you shout and hit you…if you did something good they still come and tell you it’s not good. Some teacher hates us and they put zero” (Qabelias, female 13-14 years).

### Insecurity, safety and protection

The community mapping exercise revealed where adolescents congregated and felt safe within their communities. For groups of all ages, the places perceived to be the safest were the home, school and park during the day. Mosques were also mentioned as a safe place, particularly for boys, and along with homes and dispensaries, were one of the few safe places after dark. Feelings of safety were associated with the presence of others. In drawing their homes, they referred to home as a safe and comfortable place because they were surrounded by people.

“We are safe because our parents are with us and in school our friends are there.” (Barelias, female 13-14 years)

“The home is safe because our parents and siblings are there.” (Barelias, male 15-16 years)

At the same time, the mapping revealed some unexpected fears. Places perceived to be unsafe included the streets, bakery, playground in the park, tobacco/arguile (water-pipe) shop, market, supermarket, gas station, and the park at night. Adolescent participants reported concerns for thieves, people who might kidnap, assault, beat or drug them, as well as drunk people and cars particularly at night. There were reports about verbal harassment for girls, and boys being beaten by Lebanese adolescents as they are leaving school. Female respondents talked more about kidnapping when discussing unsafe spaces than boys but kidnapping as a general concern was raised by everyone. NGOs working in the area including Save the Children had noted a recent incident of kidnapping, which may have influenced responses.

Mapping also illustrated significant gender differences experienced with regard to mobility:

“The boys are allowed to go out and the girls are not.. if they go out people will talk and start rumours” (Qabelias, female 15-16 years)

Parents expressed feeling more protective over their children – particularly their girls, now that they reside in less familiar communities. They reported that mobility of girls was restricted due to safety concerns and the perceived need to protect their reputation.

“My daughter is 12 years old and she had normal clothes on the other day when I sent her to the store. A man in a car was on the road; he kept telling her ‘come with me and we can go out.’ She got scared and since then I don’t send her outside the house” (Qabelias, adult woman)

### Changing gender norms, gender relations and family life

Most respondents confirm that cultural norms from Syria would traditionally dictate clear gender roles for men and women with fathers acting as guardians of family norms. For most of the participants, this meant that fathers tended to manage the income and rules of the household whereas the mothers managed the home. Adolescents of both genders were keenly aware of these ascribed gender roles:

“My father taught me that the man needs to go and work to bring money to the house and the women will work and clean and cook” (Barelias, male 15-16 years)

“There is a gender differentiation. Girls are supposed to work at home while the boys get home and sit in front of the TV and they expect the girl to do everything.” (Qabelias, female 15-16 years)

Adolescent girls described significant variations in how gender roles, largely dictated by their father, were experienced in their own lives. One girl described that she must leave a room when male visitors come, even in the presence of male cousins, whereas another girl shared that she would be encouraged to join a visit to another family, even when the visit involved young men (Barelias, female 15–16 years). These differences were reported to be largely dictated by the father. However, parents and adolescents described the many ways in which family relationships and gender roles were changing as a result of forced displacement from Syria. Girls described how their brothers had become more controlling and that they restricted their movements. As one girl described:

“Brothers also started to interfere and they are more strict than the parents: they put more pressure on their sisters and won’t allow them to do anything.” (Qabelias, female 15-16 years

“My brother…when he turned 20, he started telling me why you opened the door for the tea delivery guy. You can’t go out. You can’t use the phone” (Barelias, female 15-16 years)

Mothers even spoke about the power that sons have over their daughters:

“I say no [to her movement outside the home, because]…if her older brother comes and asks about her and knows that she went to the dispensary, he will wait until she returns and start beating her up! So in this case, should I let her go or not?” (Qabelias Adult woman )

Mothers in particular, expressed a strong sense of how the more liberal gender norms of Lebanese society are influencing their children. As one woman expressed it:

“The situation now is worse than in Syria. We are afraid that our kids would do something wrong.” (Qabelias, adult woman).

Another mother shared:

“Lebanese and Syrian girls, now they are harassing boys as well on whatsapp and on the road they talk to the boy and they call for him” (Qabelias, adult woman).

Women expressed how much their role as women and mothers had changed since leaving Syria’s government-supported social welfare system.

“In Syria we didn’t work and our status was better than here. We didn’t have to worry about rent and electricity and schools were for free.” (Qabelias, adult woman).

These changes deeply affected adolescent girls some of whom also reported increased involvement in the workforce since displacement.

“I work at the factory and all the women who work with me also do housework. I am proof. My dad is home…They mostly rely on me. Although I have brothers, one of them works and the other one works sometimes. I go out to work at 7 am and return at 5 or 6. When I come home, my mother asks me to do the housework” (Qabelias, female 15-16 years)

Men were also conscious of how gender roles had shifted, and in particular how limits on their income-earning potential challenged their role as male bread-winner. As one man in Qabelias put it:

“It depends on the money; if I can’t provide for my wife then I will lose her respect and we have 7 kids living in one apartment.” (Qabelias, adult man).

Women also spoke about how the respective roles of mothers and fathers had changed due to displacement. As one woman described it,

“There is a big barrier between the father and the kids. You feel that the father is escaping from the responsibility.” (Qabelias, adult woman).

Additionally, despite their increased fears, parents described being strained and less available to their children:

“Before the crisis the parents used to be more involved with their kids’ lives but now the father has many other things to worry about and the mother always worries how she will be able to provide for her kids” (Qabelias, adult woman)

Crowded households also contributed to changing family dynamics and the compromising of traditional boundaries. Men raised worries about their marital relations being observed by their children and the implications. As one man described:

“Anyway we are all living in one room it’s not right to have a relationship with my wife. And the girls should be separated from the boys” (Qabelias, adult man).

Likewise a woman in Barelias stated:

“Before each child had his own bed, the girl slept in a room alone. Now everyone is sleeping in the same room.” (Barelias, adult woman).

She also stated:

“I can’t change my clothes in front of my kids and me and my husband, we don’t have privacy anymore.” (Barelias, adult woman)

### Growing up too fast and perspectives on age appropriate behavior

Although not prominent in the narrative of adolescents themselves, parents were concerned that their children were not enjoying the childhood they could have had in Syria. Men talked about boys growing up too fast, assuming responsibilities they would not have had as children of that age in Syria and confronting new social challenges. As one man described it:

“Everyone is working to provide the rent for the house. Even my kid is 12 years old and he is asking me how much is the rent and if I was able to collect the money for it. This kid is no more a kid; he is a man.” (Qabelias, adult man).

Another man in Qabelias described how the difficult relations with the Lebanese made their sons grow up quickly, and that they want to defend their family. As he described it:

“My son is 11 years old and he went and bought a knife because the neighbor’s kid is insulting his father.” (Qabelias, adult man)

Mothers also referred to the fact that on the one hand, they are very protective of their daughters and concerned about the implications of “boys looking at her” but on the other hand, the consequence of being confined to the home and having to assume responsibilities at home meant that they are more mature than they would be at a comparable age in Syria. As one woman described this:

“I’m very strict with her. She can hold the responsibility of the house and her siblings; she can cook and clean; she is 13 but acts like a 20 year old.” (Barelias, adult woman)

At the same time, there was a strong sense of there being perceptions of socially defined and appropriate ages at which girls should be told they can no longer play with boys. However, this age varied among respondents, from as young as 7 to even 14. For example a woman in Barelias stated:

“Girls from the age of 7-8 -- you need to start tell her what is right and what is wrong, you need to say that playing with boys is not right.” (Barelias, adult woman).

### Early marriage and the changing context of marriage

Both adult and adolescent participants spoke of an observation that the appropriate age for marriage had also changed since coming to Lebanon, and respondents were often acutely aware of these changes. The sentiments of very young adolescents in relation to early marriage emerged most strongly when they were presented with a picture of a young girl holding a baby. Most young adolescents expressed their worries related to her age. They referred to her as a young child and identified with her. Some mentioned that the girl depicted in the picture is sad because she has to bear the responsibility of parenthood at an early age while she herself needs to be parented.

“She is like us but now she will grow. She is more aware of life she won’t go to school anymore” (Qabelias, female 10-12 years)

“She can’t go out and play, she is responsible for a baby” (Qabelias, female 15-16 years)

“She lost her childhood she should carry the responsibility and her life will change” (Barelias, female13-14 years)

There was widespread perception among adult participants in particular that change in the age at marriage was specifically due to new circumstances and hardships of life in forced displacement. However, there were counter views, with some expressing that people like themselves should resist these pressures. As one woman stated:

“Even if we are refugees, our children need to be mature when they get married, they will be opening and running a house.” (Barelias, adult woman)

A striking finding from focus groups with men was the awareness on the part of some participants that early marriage was not desirable, but rather a difficult choice made in difficult conditions. As one man expressed it:

“In Syria it was different girls used to marry after university and the girls who are not studying get married at 17-18” (Qabelias, adult man).

As one man described how Syrian traditions were under strain in referring to the marriage of his daughter:

“When she finished high school and a good proposal comes along it was not a problem. But now nothing allows us to keep our traditions” (Qabelias, adult man).

The discussion around early marriage needs to be seen in the context of wider changes in the nature of marriage itself that were reported by participants. A prominent theme among both the Syrian adolescent refugee girls and their parents was that the traditions associated with marriage in Syria are changing among refugees in Lebanon. People spoke of there no longer being a happy occasion with celebration, and that the expectations of marriage had been lowered and thus marriage had become less expensive and easier. Statements were typical of these sentiments such as:

“There is a man who couldn’t celebrate his son’s wedding even though he promised him a big wedding.We can’t do the wedding we wanted for our kids” (Qabelias, adult man)

Women in Barelias made statements such as:

“Now we tell the suitor 'just take her even with only a wedding band'. ”

“Marriage is so easy now: 'don’t buy her [the bride] furniture, don’t buy her anything'. ”

Women also stated that while marriage in Syria meant having a home and hopes, now it was primarily for protection of the girl. As one mother described it:

“The parents who have a daughter they want to protect her reputation [“*yesterha*” from “*sutra*” in Arabic – protecting the girl’s reputation]. Now we are refugees and when you have a daughter you think to yourself that marriage will provide her with a man to protect her and relieve us from the responsibility.” (Barelias, adult woman).

Marriages were also reported to be breaking down. A woman in Barelias stated:

“Marriage is happening in one second and divorce is happening in one second.” (Barelias, adult woman)

### Stress of displacement and mental health

Psychological stress was raised by young adolescents as sadness, depression, crying and fear. A few respondents who spoke of coping said that crying, seeing friends, taking walks, reading books, going to the sheikh, or going to school, were ways of managing sadness. One adolescent girl mentioned cutting herself. Fathers agreed that families were deeply affected by the conflict—“every house had a martyr”, and that people’s psychological wellbeing is generally bad.

The help of the mosque in improving mental health was reported by both adolescents and adults. Adolescents stated that religious activity was comforting. As one girl stated:

“When you read the Quran you feel safe.” (Barelias, female 13-14years)

Girls as young as 13–14 described the peace they felt when going to the mosque or in reading the Quran:

“I’ve been to the mosque and I really like it there. I feel good there” (Barielas, female 13-14 years)

The overwhelming theme from the focus group discussions with mothers was the enormous stress they experience out of concern for the well-being of their children and feeling they cannot address their needs. The pressures of poverty due to unemployment, underemployment and debt associated with their displacement status, were immense. Although women did not frequently discuss fears about their chidren’s mental health, it was alluded to, and particularly how images of the war in Syria could recall trauma. One woman described how:

“My daughter saw something on the TV and she was scared she cried and told me ‘don’t put the news on anymore’.” (Qabelias, adult woman)

Above all, however, women described the stress of having ultimate responsibility for their children but not being able to meet their needs or desires. One woman complained that her son did not ask anything from his father, but only of her, and another described:

“My son comes and ask me for money I tell him I don’t have any he becomes upset and start cursing then he leaves the house and we go look for him…” (Qabelias, adult woman).

Adolescent participants alluded to this stress among parents and its impact on them, although not always expicitly. There were also repeated references to the role of praying and of going to the mosque as relieving this stress. One girl in Qabelias described how,

“When our parents go to the mosque to pray they change their behaviours they become calmer and don’t shout.” (Qabelias, female 13-14 years).

Others mentioned that the mosque is a relaxing place, and that their parents forget about their problems while in the mosque.

### Health, body change and information

When asked about health and health concerns, there was a widespread perception that the poor health of the Syrian refugees was linked to their poor living conditions. Parents and adolescents pointed to poor housing, poor water supplies, lack of ventilation or natural day light as sources of their health problems. Some also pointed to crowded living conditions as not being conducive to health. Participants generally expressed favorable attitudes about existing primary health care services, and both adults and adolescents trusted local doctors at these centers as a source of information on reproductive health.

Very young adolescent respondents seem to have limited understanding of puberty except for mentioning changes like hair growth and breast growth. Many said they would like to have more information. Laughing was a common response to these questions as well as shyness, and this was even the case among those in the 15–16 age group. Adolescents mainly acquired information through television or mobile technology, and were embarrassed to ask their mothers for information. Some girls described their sisters as being the best source of information rather than their mothers. Interestingly, however, several mothers insisted that parents should be the primary source of information regarding bodily changes. One mother stated clearly that she tells her girls not to play with boys but not why:

“It’s not allowed to play with boys but we don’t tell a girl why it’s not allowed. We don’t make her aware of these things.” (Qabelias, adult woman)

Some expressed concern about their girls getting inappropriate knowledge from television and technology. However, several stated that it was only appropriate to give them such information at puberty, or at the age of 12 or even just before they marry. In other cases, however, mothers recognized that they themselves needed more information and if they had it, they could talk in a better way with their children. Others expressed relief that their daughters were learning from technology as at least they were getting information that as parents they found difficult to share with them.

At the same time, men expressed recognition that since their children were growing up more quickly, they also needed information. As one man described it:

“In these settings, the 14-15 years old kids are more aware of marriage because we are all living in one room and the kid feels that there is something between the father and the mother” (Qabelias, adult male)

## Discussion

This qualitative study, one of the first among Syrian refugee adolescents in Lebanon, revealed perceptions about disrupted young lives, with reduced access to education as compared to almost universal enrolment in Syria, concerns over family economic situations and changing gender roles and family relationships. Constraints to mobility and parental fears about their children’s behavior in the new setting in which they found themselves seems to have affected girls much more than boys. Parents were also concerned about the exposure – particularly of adolescent girls - to the more liberal social norms of Lebanese society as their children matured into adolescents in an unfamiliar environment. Mothers emphasized the stress they experienced trying to make ends meet and provide for their children with unemployed or underemployed husbands.

Adolescents are particularly vulnerable in such situations, passing through a difficult period of life in need of family and educational opportunities, and parental stress decreased their capacity to support their adolescent children. Adolescents participating in the study were preoccupied by family economic concerns and shortfalls in their education. They experienced social exclusion in schools and community life despite sharing a common culture and language with the host country.

As more Syrian refugee adolescents in Lebanon are enrolled in already under-sourced public schools, with a major drive by the Ministry of Education to increase their enrolment, there is potential for school-based interventions to promote mental health as has been attempted in other conflict-affected adolescent populations in Burundi [14] and Nepal [15]; moreover within the same region as this study, an example of an intervention for 10 to 12 year old Palestinian children affected by conflict has shown promise in terms of improving psychosocial well-being there [16].

For very young adolescents both in and out of school, a number of NGOs working in Jordan and Lebanon have introduced child and adolescent-friendly ‘safe spaces’ to protect them from risk and promote their psychosocial health. Internationally, however, these have not been suffeciently evaluated in humanitarian settings [17]. One evaluation in Jordan, however, found that while some of the objectives were achieved, programming needed to be improved to address psychosocial well-being, and that the safe spaces worked betteer for younger children than adolescents [18].

Given the evident stresses that parents, and particularly mothers, are experiencing in this context, potential support for parenting in the context of displacement needs to be explored. There is a potential role here for religious leaders given the comfort all reported from going to the mosque and praying. Economic empowerment and vocational training for both older adolescents and their parents would help to reduce the financial stresses created by their forced displacement that has implications for mental health risks of both parents and adolescents. There are a limited numbers of sectors where Syrian refugees are authorized to work – including agriculture, construction and domestic work (Decree 197 of the Ministry of Labour, implemented in December 2014) – and therefore opportunities for increased engagement in those sectors on a non-coercive basis need to be explored.

### Limitations

The short duration of time for the fieldwork for this study made it difficult to explore in detail the differences between the two settings and between the experience of adolescent Syrian refugees and adolescents in the host communities. Further research on the latter is particularly needed given the long-lasting nature of the forced displacement from the Syrian crisis, the competition over employment in Lebanon and the tensions voiced by respondents. A further constraint is that there is a very limited literature on adolescents’ experiences in Syria prior to the conflict with which to compare the findings; and therefore it is difficult to discern if findings are specific to the refugee setting or to the cultural context.

## Conclusion

The study points to the need for special attention to this age-group of adolescents 10 to 14 years of age in the humanitarian response to the Syrian forced migration crisis. In particular, there is an urgent need to increase educational access for adolescents 13 years old and above, only a minority of whom were enrolled in school at the time of the study, and to address school drop-out. Special attention is needed to gender differentials in factors encouraging enrolment and leading to drop-out, such as security concerns for girls. The study also found that Syrian adolescents experience tensions with Lebanese adolescents and therefore further programmatic efforts to address integration and mutual respect are needed. Lebanese teachers need to be trained and sensitized about the problems experienced by adolescent Syrian refugees and on the need for better integration with Lebanese adolescents.

Finally, our study points to the need for adolescents to have better access to sexual and reproductive health information and the need to address the special risks posed by early marriage. Above all, as has been confirmed by earlier studies [19], there is a need for greater evidence on effective interventions for conflict-affected very young adolescents.
